# Subgroups of Foot-Ankle Movement Patterns Can Influence the Responsiveness to a Foot-Core Exercise Program: A Hierarchical Cluster Analysis

**DOI:** 10.3389/fbioe.2021.645710

**Published:** 2021-06-08

**Authors:** Ricky Watari, Eneida Y. Suda, João P. S. Santos, Alessandra B. Matias, Ulisses T. Taddei, Isabel C. N. Sacco

**Affiliations:** Department of Physical Therapy, Speech and Occupational Therapy, Faculdade de Medicina, Universidade de São Paulo, São Paulo, Brazil

**Keywords:** running, foot, ankle, cluster analysis, principal component analysis, foot-core, exercise therapy, biomechanics

## Abstract

The purpose of this study is to identify homogenous subgroups of foot-ankle (FA) kinematic patterns among recreational runners and further investigate whether differences in baseline movement patterns can influence the mechanical responses to a foot-core exercise intervention program. This is a secondary analysis of data from 85 participants of a randomized controlled trial (clinicaltrials.gov – NCT02306148) investigating the effects of an exercise-based therapeutic approach focused on FA complex. A validated skin marker-based multi-segment foot model was used to acquire kinematic data during the stance phase of treadmill running. Kinematic features were extracted from the time-series data using a principal component analysis, and the reduced data served as input for a hierarchical cluster analysis to identify subgroups of FA movement patterns. FA angle time series were compared between identified clusters and the mechanical effects of the foot-core exercise intervention was assessed for each subgroup. Two clusters of FA running patterns were identified, with cluster 1 (*n* = 36) presenting a pattern of forefoot abduction, while cluster 2 (*n* = 49) displayed deviations in the proximal segments, with a rearfoot adduction and midfoot abduction throughout the stance phase of running. Data from 29 runners who completed the intervention protocol were analyzed after 8-weeks of foot-core exercises, resulting in changes mainly in cluster 1 (*n* = 16) in the transverse plane, in which we observed a reduction in the forefoot abduction, an increase in the rearfoot adduction and an approximation of their pattern to the runners in cluster 2 (*n* = 13). The findings of this study may help guide individual-centered treatment strategies, taking into account their initial mechanical patterns.

## Introduction

The foot-ankle complex (FA) is an important structure for supporting and dissipating the weightbearing forces that are applied to the body, especially during dynamic locomotor skills, such as gait. The FA performs the role of load absorption and propulsion by means of the deformation of its viscoelastic structures, which can be either passive, such as the plantar aponeurosis (Ker et al., 1987), or active tissues, among which, the intrinsic muscles have an important participation for functioning as an active spring-damper for energy dissipation and production (Kelly et al., 2018; Riddick et al., 2019).

The function of the foot-core system and the potentially beneficial effects of its training have been receiving increasing attention in the clinical and athletic fields because of the critical role of the intrinsic foot muscles as local stabilizers and direct sensors of foot deformation (McKeon et al., 2015). Increasing loads on the foot causes a progressive lowering of the medial longitudinal arch, and the activation of intrinsic foot muscles is able to control and even counteract such deformation, by stiffening the arch structure, suggesting important implications for how forces are transmitted during locomotion and postural activities (Kelly et al., 2014). In a study addressing the link between falls among older people and foot deformities and intrinsic foot-muscle weakness (Mickle et al., 2009), it was identified that each unit (% body weight) increase in hallux strength had the potential of decreasing the odds of sustaining a fall by 7%. Furthermore, a 12-week foot-core training program resulted in significant increases in hallux and lesser toe strength, along with improvements in single leg stance balance in this population (Mickle et al., 2016). In a study seeking to improve athletic performance, the jump performance of young athletes significantly improved after isometric training of the hallux flexion for 7 weeks (Goldmann et al., 2013). Thus, there is evidence in the literature regarding the benefits of strengthening the foot-core muscles for general body function and balance. Moreover, during running, wherein the motor behavior is more dynamic, the function of the FA is even more important, with evidence of its influence on running-related injury prevention. Taddei et al. (2020a) showed that a foot-core exercise intervention program in middle and long-distance recreational runners resulted in 2.4 times lower injury incidence when compared to a control group in a 12 months period.

However, distinct individuals might show different responses to the same exercise-based therapeutic approach, which can be related to differences in biomechanical patterns or motor adaptations. For example, Kobsar et al. (2015) identified that among individuals with knee osteoarthritis, there were subgroups that presented different levels of response to the proposed hip and knee strengthening exercises. The high-responder group, who displayed greater levels of improvement in pain and function, had higher degrees of hip adduction during the load response phase of gait than the other subgroups at baseline. Asymptomatic runners have been reported to present subgroups of distinct hip, knee and ankle kinematic patterns (Phinyomark et al., 2015). The presence of distinct subgroups with homogeneous running gait patterns have also been described among runners with different injuries, although the described patterns were not related to injury location (Jauhiainen et al., 2020), and individuals with the same type of injury can exhibit different kinematic patterns (Watari et al., 2016; Dingenen et al., 2020). However, this type of analysis was not extended to the FA complex. Given that the FA is a multisegmented structure, it is possible that different movement combinations could occur within that complex structure with more than 30 joints and 25 muscles during running and influence the effects of an exercise intervention.

Therefore, the aim of this study is to identify homogenous subgroups of FA kinematic patterns among recreational runners and further investigate whether differences in baseline movement patterns can influence the mechanical responses to a foot-core exercise program.

## Materials and Methods

The present study is a secondary analysis of a 12-month randomized single-blind parallel controlled trial, registered prospectively at clinicaltrials.gov (NCT02306148), which aimed to investigate the effects of an exercise-based therapeutic approach focused on the FA complex over the incidence of lower limb running-related injuries in middle and long-distance recreational runners. This secondary analysis used data from the baseline and week-8 (post-intervention) assessment time points.

### Participants and Recruitment

The methods of this randomized controlled trial have been previously published in detail by Matias et al. (2016) and are briefly described here. The study was approved by the Ethics Committee of the School of Medicine of the University of São Paulo (CAAE 41171215.7.0000.0065) and prior to participation, all runners gave written informed consent. A total of 118 recreational runners were recruited in the original trial and were randomly allocated to either an intervention (*n* = 57) or a control group (*n* = 61). The included runners were between 18 and 55 years old, who had been running for at least 1 year, at least 20 km per week, and no more than 100 km per week, with no running-related injuries in the 2 months prior to baseline assessment, no experience running barefoot or in minimalist shoes, and without chronic diseases or impairments that could influence running performance. To ensure a generalizable sample, runners were recruited from the local community, through digital social media advertising, direct contact with running groups in the university surroundings, and word of mouth.

Data from 85 participants were included for this secondary analysis, based on the availability of the whole time series of FA kinematic data of at least 10 step cycles in the baseline assessment. To investigate the effect of the foot-core intervention in the clusters found at baseline, 29 runners who completed the intervention protocol were analyzed.

### Foot Core Intervention

Runners in the intervention group received an 8-weeks program of foot-core muscle training containing 12 exercises that progressed weekly in volume and difficulty (Matias et al., 2016). The training was performed once a week with a physiotherapist and participants received online access to the exercise descriptions and videos (web-software) to perform them with an additional of 3 × /week, remotely supervised by the same physiotherapist. Participants in the control group were instructed to perform a 5-min placebo static stretching protocol 3 × /week based on online descriptions (web-software) and images (Matias et al., 2016; Taddei et al., 2020b).

All subjects had a biomechanical evaluation at baseline and after 8 weeks, and injuries were continually assessed throughout the study by a web-software for 12 months. Running-related injuries were defined as “any musculoskeletal pain or injury that was caused by running practice and led to changes in the form, duration, intensity or frequency of training for at least one week” (Macera et al., 1989).

### Biomechanical Data Acquisition and Processing

Participants were asked to run barefoot at a self-selected speed on an instrumented treadmill at ground level, embedded with two force plates in tandem position (AMTI Force-Sensing treadmill AMTI, Watertown, EUA; force plates at 1000 Hz). They were asked to maintain their self-reported footstrike pattern. FA kinematics were acquired using eight infrared cameras (Vicon^®^ VERO, Vicon Motion System Ltd., Oxford Metrics, United Kingdom; at 200 Hz), with 16 reflective skin markers (9 mm in diameter) placed according to the Rizzoli Foot Model (Leardini et al., 2007; Portinaro et al., 2014). A 2–3 min warm-up and familiarization period, while running barefoot on the treadmill, was given to each participant, after which, kinematic data was recorded for 30 s in order to acquire at least 10 step cycles for each limb. Only data from the dominant limb was used in the analysis, which was determined as the one with which the subject would kick a ball (Zifchock et al., 2006).

The Nexus software (version 2.10.3, Vicon, Oxford, United Kingdom) was used to reconstruct the 3D coordinates of the skin markers during running. Marker trajectories were filtered using a Woltring low-pass filter (cutoff frequency = 10 Hz), and processed in Visual3D (C-Motion, Germantown, MD, United States) for joint angle calculation using the joint coordinate system (Wu et al., 2002). The multisegmental foot model utilized in this study also followed the joint coordinate system according to ISB recommendations (Leardini et al., 2007). Accordingly, the following convention for joint rotations was established: dorsi/plantarflexion was assumed to be the rotation about the *Z*-axis (medio-lateral) of the proximal segment, abduction/adduction the rotation about the *Y*-axis (vertical) of the distal segment, and eversion/inversion the rotation about the axis orthogonal to the previous two. Ground reaction forces were sampled at 1000 Hz and used to determine stance phase, which was defined using a 10 N threshold and normalized in time to 101 points (0–100% stance phase).

Joint angle time series in all three planes were extracted from the following: angle between shank and calcaneus (Sha-Cal), calcaneus and midfoot (Cal-Mid), and midfoot and metatarsal bones (Mid-Met). The footstrike angle was defined as the sagittal-plane foot angle relative to the ground at foot contact. Positive foot angles were classified as rearfoot strike pattern and negative angles as forefoot strike pattern (Matias et al., 2020).

### Cluster Analysis Procedures – Baseline Data

The kinematic time series from baseline assessment of the 85 runners were averaged across steps for each subject and combined into a 909-dimensional row vector for each participant (101 data points per axis direction for each joint kinematic waveform), creating an 85 × 909 data matrix (85 runners × 909 data points). The data matrix was assessed by an algorithm for outlier detection in multivariate samples (Trujillo-Ortiz, 2020), which found no outliers. The data was standardized to a mean of 0 and standard deviation of 1 (Kettaneh et al., 2005), after which a principal component analysis (PCA) was applied for data reduction and feature extraction, given the large number of dependent variables and potential for data redundancy. PCA is an orthogonal transformation technique used to convert a set of variables into a set of linearly uncorrelated variables by determining new bases called principal components (PCs) that maximize the variability in the original data set (Abdi and Williams, 2010).

We retained only the PCs that contained up to 99% of variance explained, which underwent a hierarchical cluster analysis (HCA) to identify homogeneous foot-ankle movement patterns during running. The PCs were used in the HCA to avoid collinearity of the input data and to enable the identification of multi-segment patterns. The HCA created a cluster tree (or dendrogram) using an agglomerative strategy, or a “bottom-up” approach, which consists of three steps: (1) a measure of dissimilarity between sets of subjects using the Euclidean distance, (2) subject linkage using the Ward’s minimum variance method (Ward, 1963), and (3) optimal cluster determination based on the cluster division that rendered the highest average Silhouette index (Rousseeuw, 1987; Arbelaitz et al., 2013; Nisha and Kaur, 2016).

The Silhouette index measures the quality of the cluster solution based on the average distance between one element and all the others in the same cluster, and the average dissimilarities with all the objects outside the cluster, resulting in a silhouette width ranging between −1 and +1. A negative value indicates that the element does not belong to the assigned cluster, and a positive value represents the degree of belonging to the assigned cluster. The quality of grouping was also evaluated with the cophenetic correlation coefficient (CCC) to verify the internal validity of the clustering (Sokal and Rohlf, 1962; Carvalho et al., 2019). The CCC measures the degree of fit between the observed distances between the elements and the distances predicted from a grouping process, resulting in a coefficient ranging from 0 to 1, wherein the closer to 1, the better the grouping quality (Carvalho et al., 2019), and a CCC lower than 0.7 indicates a grouping method with low internal validity (Rohlf, 1970).

Following identification of homogeneous clusters of runners, the determined subgroups were compared for differences in demographic and training characteristics, and baseline PC scores representing foot-ankle patterns, by means of independent *t*-tests for continuous variables and χ^2^ tests for categoric variables (α = 0.05). The Cohen’s *d* effect sizes and 95% confidence interval of the differences in baseline PC scores between clusters were also calculated. Additionally, the PCs that presented significant differences between clusters were further assessed for interpretation of the kinematic pattern they represented, by inspection of the shape of the PC loading vector, the differences between representative extremes (5th and 95th percentile PC-scores), and the single component reconstruction of the 5th and 95th percentile PC-scores (Brandon et al., 2013).

A qualitative analysis of the mean baseline kinematic time series across clusters was performed, based on the interpretation of the biomechanical representation of the PCs that had significant differences. The PC loading vector indicates the regions of the time series dataset where each component has high or low representation according to the variance of the data in that particular rotated dimension. The differences between the raw waveforms of representative subjects of the extreme ends of the PC-score scale can be used to understand the differences between high and low-scoring individuals, specifically in the regions where the PC loading vector has high values. This way, the observed differences can be attributed to the PC of interest, excluding the additional inter-subject variance captured by the other PCs. The specific biomechanical effects represented by the PC of interest can be further visualized by plotting the upper and lower bands about the mean waveform, by reconstructing the signal using only the coefficient of the target PC and the representative scores for the 5th and 95th percentile and comparing those extremes (Brandon et al., 2013).

### Effects of the Foot-Core Intervention – Baseline vs. Week-8

Only the runners in the intervention group that completed the protocol and attended both baseline and week-8 assessments were included in this portion of the analysis, resulting in 29 subjects. The running mechanics of the intervention group was compared before (baseline) and after (week-8) the foot-core exercise program to better understand the influence of the baseline foot kinematic pattern on the response to the intervention. This was an exploratory analysis to provide some insight into the role of biomechanical subgroups on the response to an exercise-based approach.

Kinematic waveforms from the week-8 assessment were transformed into the PC space of the initial PCA, and a 2 × 2 mixed model ANOVA (Tukey *post hoc* test) considering the different clusters and assessment times as the between and within-factors, respectively, was applied to the 16 PC scores that were used as inputs to the HCA (α = 0.05). The partial η^2^ of the interaction effect and the 95% confidence interval of the between and within-group differences were also calculated.

A further qualitative analysis of the kinematic time series was performed, only regarding the changes related to the PCs with significant interaction effects, to better understand the biomechanical responses to the intervention in each foot-ankle movement pattern subgroup.

All data processing and analyses were performed in MATLAB R2015a (MathWorks, Natick, MA, United States) and statistical tests were applied using Statistica 13.5 (TIBCO Software Inc., Palo Alto, CA, United States).

## Results

### Identification of Subgroups (Clusters) at Baseline

**FIGURE 1:**
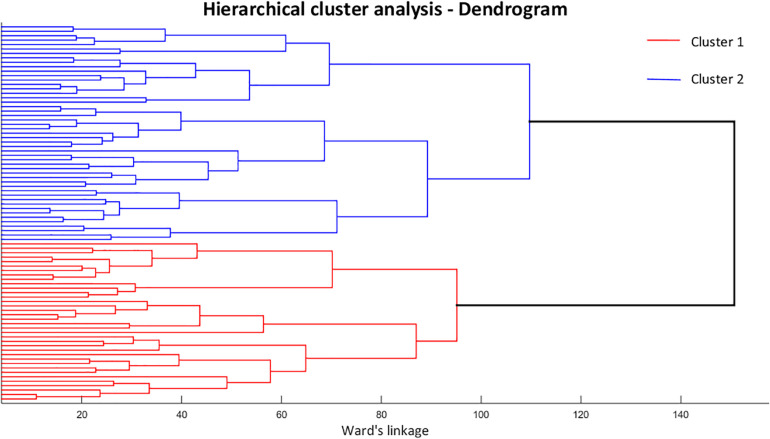
Dendrogram resulting from the hierarchical cluster analysis, representing element combinations and dissimilarity level for each pairing. Cluster 1 is represented by the red lines and cluster 2 by the blue lines.

The optimal cluster division was determined as a solution with two clusters (Figure 1), achieving an average Silhouette index of 0.13 and a CCC of 0.38. The characteristics of the subjects are presented in Table 1. The first (*n* = 36) and second clusters (*n* = 49) did not present significant differences between them with respect to demographic and running practice characteristics, or incidence of injuries by the end of the clinical trial, but there were proportionally more forefoot strikers in cluster 2 (49%) when compared to cluster 1 (25%).

**TABLE 1:** Mean (SD) and distribution of demographic and running practice characteristics, and number of injured runners by the end of the clinical trial.

	All (*n* = 85)	Cluster 1 (*n* = 36)	Cluster 2 (*n* = 49)	*p*	95% CI
Age [years]	40.4 (7.0)	39.9 (6.0)	40.7 (7.6)	0.618^a^	[−3.8; 2.3]
Height [m]	1.69 (0.09)	1.69 (0.09)	1.69 (0.09)	0.903^a^	[−0.04; 0.04]
Body mass [kg]	70.3 (13.1)	69.7 (13.9)	70.6 (12.6)	0.757^a^	[−6.6; 4.9]
Body Mass Index [kg/m^2^]	24.4 (3.2)	24.3 (3.5)	24.5 (3.0)	0.721^a^	[−1.7; 1.2]
Sex [n female/n male]	42/43	17/19	25/24	0.729^b^	–
Running volume [km/week]	20.5 (13.2)	20.0 (13.2)	20.7 (13.3)	0.814^a^	[−6.7; 5.3]
Practice time [h/week]	2.1 (1.3)	2.1 (1.2)	2.2 (1.4)	0.728^a^	[−0.7; 0.5]
Pace [min/km]	6.6 (1.4)	6.6 (1.4)	6.6 (1.3)	0.958^a^	[−0.6; 0.7]
Injured in 12-months [n]	19	5	14	0.108^b^	–
Footstrike pattern [n forefoot/n rearfoot]	33/52	9/27	24/25	0.025^b^*	–

*^*a*^Independent *t*-test.*

^*b*^χ^2^ test.

*Significant difference between clusters.

Out of 84 PCs resulting from the FA kinematic time series, the first 16 PCs were retained for analysis, representing a total of 99.1% of variance explained, and they served as inputs for the HCA. Only PC1 and PC3 presented significant differences between clusters (Table 2).

**TABLE 2:** Mean (SD) and variance explained of the principal components (PCs) included in the hierarchical cluster analysis (HCA).

PCs [a.u.]	Cluster 1 (*n* = 36)	Cluster 2 (*n* = 49)	Variance explained (%)	*p*^*a*^	95% CI	Cohen’s *d*
PC1	−12.6 (9.7)	9.3 (10.3)	23.9	< 0.001*	[−26.2; −17.5]	0.39
PC2	2.4 (12.9)	−1.7 (12.7)	18.1	0.148	[−1.5; 9.6]	0.05
PC3	−3.2 (12.7)	2.3 (11.5)	16.5	0.037*	[−10.8; −0.3]	0.08
PC4	−2.1 (11.5)	1.6 (9.5)	12.1	0.111	[−8.2; 0.9]	0.05
PC5	−1.3 (8.8)	0.9 (10.4)	10.5	0.314	[−6.4; 2.1]	0.04
PC6	−0.8 (9.0)	0.6 (8.6)	8.5	0.504	[−5.1; 2.6]	0.02
PC7	−0.3 (6.2)	0.3 (5.2)	3.5	0.638	[−3.0; 1.9]	0.02
PC8	0.2 (3.9)	−0.2 (4.5)	2.0	0.655	[−1.4; 2.3]	0.02
PC9	0.2 (3.4)	−0.1 (3.6)	1.4	0.740	[−1.3; 1.8]	0.01
PC10	0.1 (3.4)	−0.1 (3.1)	1.2	0.851	[−1.3; 1.6]	0.01
PC11	−0.1 (2.2)	0.1 (2.1)	0.5	0.652	[−0.8; 0.9]	0.02
PC12	0.1 (2.3)	0.0 (1.7)	0.4	0.855	[−1.1; 0.3]	0.01
PC13	−0.2 (1.5)	0.2 (1.6)	0.3	0.223	[−1.1; 0.3]	0.05
PC14	−0.1 (1.3)	0.1 (1.2)	0.2	0.592	[−0.7; 0.9]	0.02
PC15	−0.1 (1.2)	0.1 (1.2)	0.2	0.441	[−0.7; 0.3]	0.03
PC16	0.1 (0.9)	−0.1 (1.2)	0.1	0.645	[−0.4; 0.6]	0.02

*Statistically significant differences between clusters (in bold).

*^*a*^Independent *t*-test.*

**FIGURE 2:**
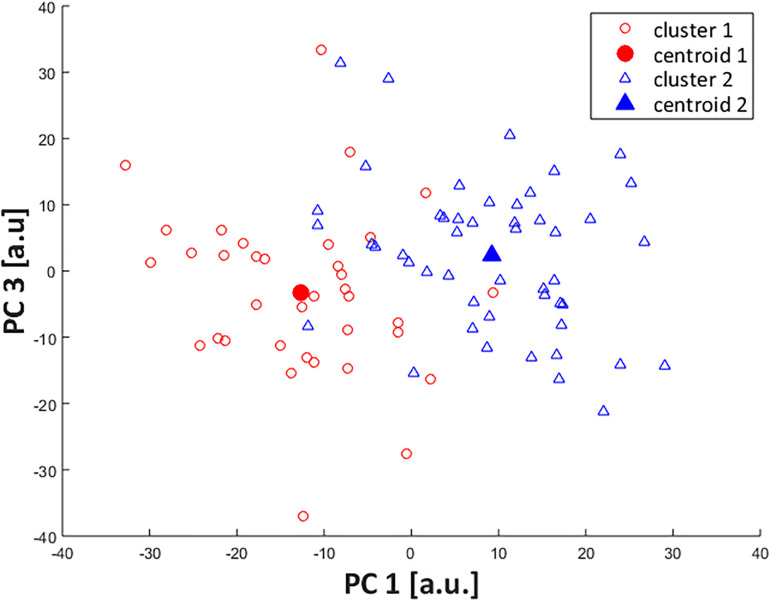
Scatterplot of PC1 and PC3 scores across identified clusters. Subjects from cluster 1 are represented by open red circles (cluster centroid as filled circle) and from cluster 2, by open blue triangles (cluster centroid as filled triangle).

The cluster grouping can be further visualized with a scatterplot of PC1 and PC3 across the two clusters (Figure 2). As shown by the statistical results, it is clear that PC1 is the feature that better depicts the separation between subgroups, in which cluster 1 presents negative values of PC1 scores and cluster 2 has more positive values, in their majority; while the distinction is less clear for PC3 scores.

### Interpretation of FA Movement Components Across Clusters at Baseline

Only PC1 and PC3, which presented statistically significant differences between the two clusters, were considered for this part of the analysis.

**FIGURE 3:**
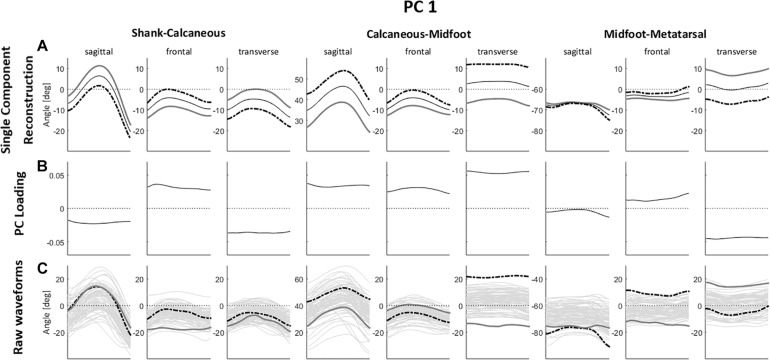
**(A)** Single component reconstruction representing only the variance due to PC1 for the 5th (gray solid lines) and 95th (black dashed lines) percentile extreme representatives, and the mean reconstructed waveform (thin black solid lines); **(B)** PC loading vector with the pattern of variance captured by PC1; **(C)** raw waveforms for the 5th (gray solid lines) and 95th (black dashed lines) percentile extreme representatives for PC1, superimposed on all raw waveforms (thin gray solid lines). Positive (+) and negative (–) joint angles represent the following – sagittal plane: (+) dorsiflexion, (–) plantar flexion; frontal plane: (+) eversion, (–) inversion; transverse plane: (+) abduction, (–) adduction.

For PC1 (Figure 3), the highest absolute values in the PC loading vector occurred in regions corresponding to movements in the transverse plane, especially for calcaneus-midtarsal (Cal-Mid) and midtarsal-metatarsal (Mid-Met) joints. Those also display the greatest differences between representative extremes of both the raw waveforms and the single component reconstruction, depicting a magnitude feature and a difference feature. High PC1 scores correspond to greater Cal-Mid abduction and shank-calcaneus (Sha-Cal) and Mid-Met adduction, and the larger the difference in direction between the midfoot and the other foot segments, the higher is the absolute value of PC1 score.

There is also a perceptible difference in the Sha-Cal frontal plane and Cal-Mid sagittal plane with a relatively high value in the PC loading vector for this region, indicating that higher PC1 scores represent lower degrees of rearfoot inversion and greater midfoot dorsiflexion. Additionally, those movement patterns are associated to a higher degree of forefoot abduction, depicted by a difference feature with positive loading coefficients for Sha-Cal frontal and Cal-Mid sagittal planes, and negative coefficients for Mid-Met transverse plane.

In summary, runners with positive PC1 scores present a less inverted rearfoot (closer to neutral), an abducted and more dorsiflexed midfoot, and an adducted forefoot during the stance phase of running; while negative PC1 scores indicate greater inversion of the rearfoot, an adducted and less dorsiflexed midfoot, and an abducted forefoot.

**FIGURE 4:**
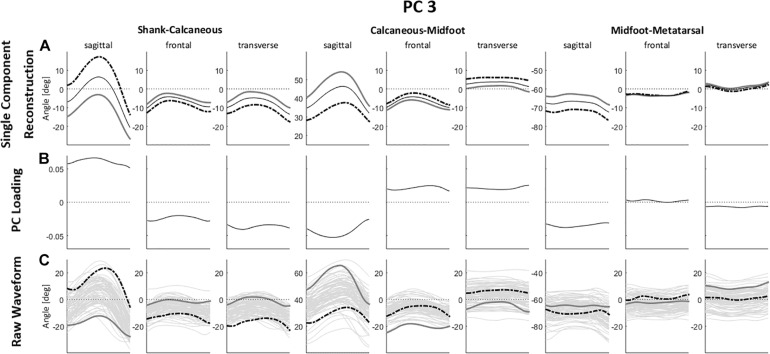
**(A)** Single component reconstruction representing only the variance due to PC3 for the 5th (gray solid lines) and 95th (black dashed lines) percentile extreme representatives, and the mean reconstructed waveform (thin black solid lines); **(B)** PC loading vector with the pattern of variance captured by PC3; **(C)** raw waveforms for the 5th (gray solid lines) and 95th (black dashed lines percentile extreme representatives for PC3, superimposed on all raw waveforms (thin gray solid lines). Positive (+) and negative (–) joint angles represent the following – sagittal plane: (+) dorsiflexion, (–) plantar flexion; frontal plane: (+) eversion, (–) inversion; transverse plane: (+) abduction, (–) adduction.

As for PC3 (Figure 4), the highest absolute values in the PC loading vector occurred in the sagittal plane of Sha-Cal and Cal-Mid joints, and both the representative extremes of raw waveforms and the single component reconstruction show that higher PC3 scores correspond to a rearfoot dorsiflexion and a lower degree of midfoot dorsiflexion throughout the stance phase. There are also relatively high values in the PC loading vector in the regions relative to Sha-Cal and Cal-Mid joints in frontal and transverse planes, with magnitude differences, in which higher PC3 scores represent greater rearfoot inversion and adduction, and an abducted midfoot with lower degrees of inversion. Additionally, a difference feature also occurs between rearfoot and midfoot transverse plane movements, where positive PC3 scores indicate a greater difference in direction between the two segments.

In brief, positive values of PC3 score point to a rearfoot in dorsiflexion, inversion and adduction; a midfoot with less dorsiflexion and inversion, and in an abducted position; in contrast to negative PC3 scores, which correspond to a rearfoot in plantar flexion, and closer to neutral in the frontal and transverse planes, and a midfoot with greater dorsiflexion, inversion and adduction.

### Differences in FA Movement Patterns Between Clusters at Baseline

The qualitative analysis of FA movement patterns across clusters was based on PC1 and PC3. However, since PC1 contained a greater level of variance explained, and showed a greater effect size in between-cluster differences (Table 2), most of the observable differences between cluster 1 and 2 were related to PC1.

**FIGURE 5:**
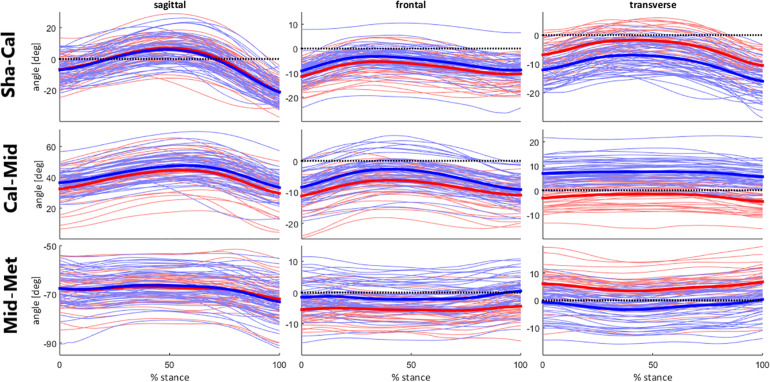
Foot segment joint angles across clusters during stance phase of running. Runners from cluster 1 are represented by the red lines; and from cluster 2, by the blue lines. Mean kinematic waveforms for each cluster are represented by the thick solid lines.

**FIGURE 6:**
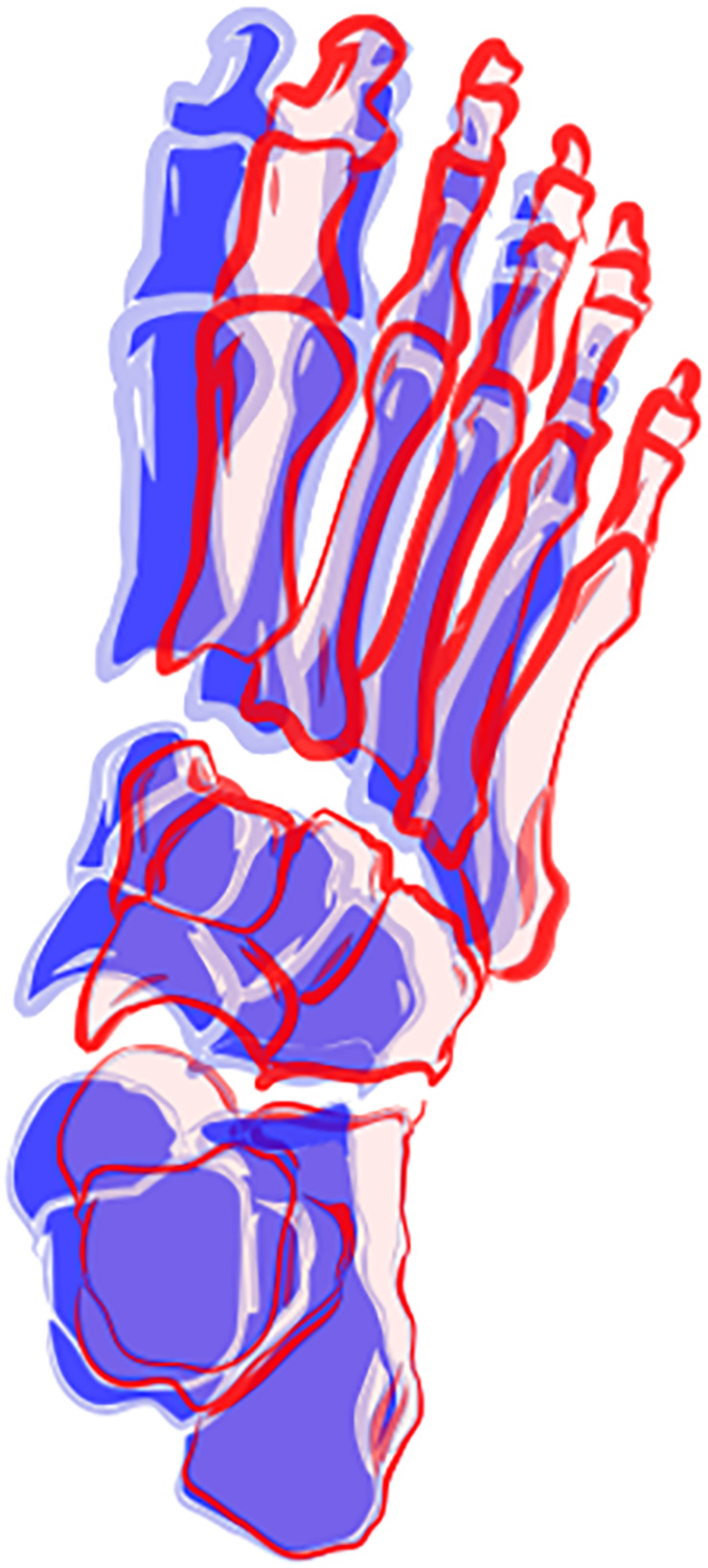
Illustration of between-cluster differences of foot and ankle movement patterns in the transverse plane. The average pattern for cluster 1 is represented in red and for cluster 2 is represented in blue.

Cluster 1 presented more negative values for both PC1 and PC3, indicating that they display a movement pattern that is more similar to the 5th percentile in both components. Runners from cluster 1 ran with a less adducted rearfoot (closer to neutral position); combined with a midfoot adduction (also closer to neutral position); and a forefoot abduction. As for cluster 2, who had more positive values for PC1 and PC3 and were, therefore, more similar to the 95th percentile, their runners presented a greater rearfoot adduction; a midfoot abduction; and kept the forefoot close to neutral position, but with a slight adduction. Differences in frontal and sagittal planes that were related to either PC1 or PC3 did not appear as clearly in the between-cluster comparison (Figure 5). The main average differences between clusters in the transverse plane can be visualized in Figure 6.

### Effects of the Foot-Core Intervention: FA Kinematic Changes Across Clusters - Baseline vs. Week-8

**FIGURE 7:**
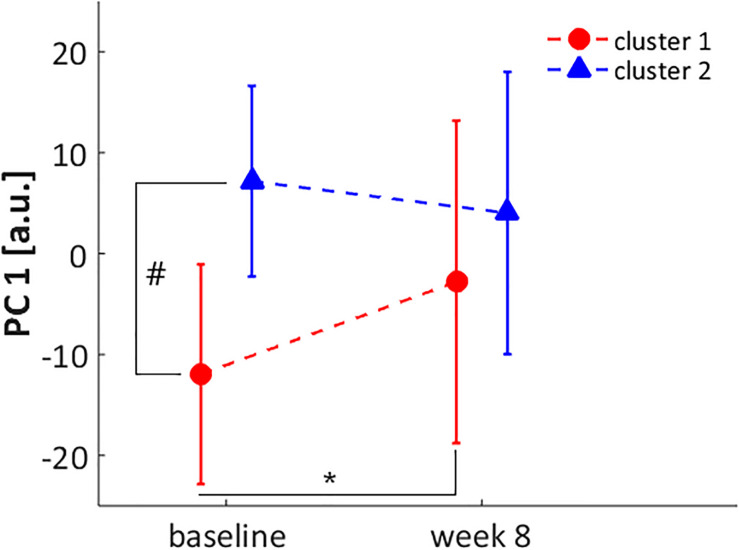
Score for PC1 at baseline and week 8 assessments for cluster 1 (red circle) and cluster 2 (blue triangle). * significant difference between baseline and week 8 assessment in cluster 1. # significant difference between cluster 1 and 2 at baseline.

Out of the initial sample (*n* = 85), only 29 participants were part of the intervention group and completed the baseline and week-8 assessment after the exercise protocol (16 participants in cluster 1 and 13 in cluster 2). The only PC that presented a significant cluster X assessment time interaction was PC1 (*F* = 7.66; *p* = 0.010; $\eta_{p}^{2}$ = 0.22), wherein the clusters were significantly different at baseline [mean difference = 19.1; 95% CI = (5.4; 32.9)], but only cluster 1 presented a significant change after the foot-core exercise protocol [mean difference = 9.2; 95% CI = (0.7; 17.6)], according to the *post hoc* test (Figure 7).

**FIGURE 8:**
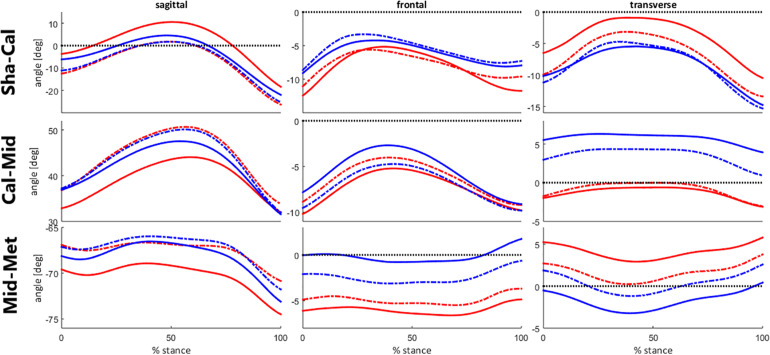
Foot segment joint angles across clusters during stance phase of running, before (baseline) and after the intervention protocol (week-8). Runners from cluster 1 are represented at baseline by the red solid lines and at week-8 by the red dashed lines. Runners from cluster 2 are represented at baseline by the blue solid lines and at week-8 by the blue dashed lines.

**FIGURE 9:**
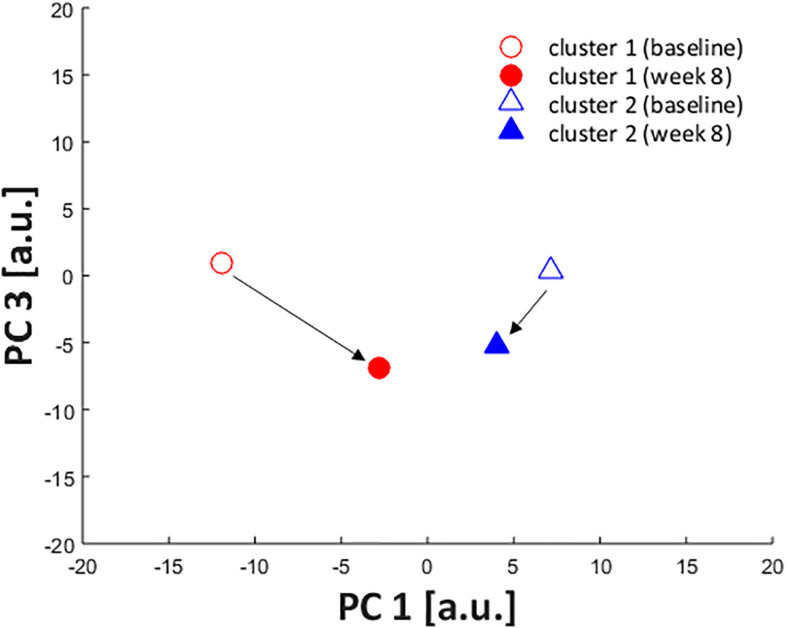
Scatterplot showing PC1 and PC3 scores of cluster centroids at baseline (open) and week 8 (filled) assessments. The centroid of cluster 1 is represented by red circles and cluster 2, by blue triangles.

Considering the kinematic features represented by PC1, after the intervention, runners from cluster 1 presented an increase in rearfoot adduction and a decrease in forefoot abduction. Additionally, there was an increase in Cal-Mid dorsiflexion, which, added to the changes in transverse plane, made their movement pattern more similar to cluster 2 (Figure 8). The approximation of movement patterns regarding PC1 can be confirmed in the scatterplot (Figure 9), where it is possible to observe a shift in the centroid position of cluster 1 towards the centroid of cluster 2 in the PC1 dimension.

## Discussion

The present study aimed at performing an exploratory analysis to identify homogenous subgroups of FA movement patterns in recreational runners, and examine whether such patterns could be influencing the biomechanical responses to a foot-core exercise intervention. With the use of an HCA, two clusters of FA joint kinematics were identified, wherein the runners displayed differences predominantly in the transverse plane of all three-foot segments.

Although a two-cluster division was considered the optimal cluster solution, it resulted in a low clustering quality in both the average silhouette index (0.13) and the CCC (0.38), indicating that the identified subgroups may be too heterogeneous to define clear differences in FA movement patterns, which can be confirmed by the lack of significant differences in most of the PC scores at baseline. Therefore, the results reported in this study must be considered with caution, since the indicators of cluster quality are very low and limits the generalizability of our findings. However, the clusters were distinct regarding PC1 and PC3 and the scatterplot across them (Figure 2) shows a spatial divergence, verifying the validity of the defined subgroups. Together, both of the PCs presented 40.4% variance explained by the FA joint kinematics (Table 2), with PC1 having the largest effect size between clusters. Since PC1 had a greater relative representation of midfoot and forefoot transverse plane movements (Figure 3), it is clear that this component drove most of the differences in movement patterns.

The overall difference between subgroups occurred around the segment that was mainly deviated from neutral position in the transverse plane during the stance phase. On average, cluster 1 displayed a greater Mid-Met abduction, while cluster 2 presented greater deviations in the more proximal foot segments, with a more adducted Sha-Cal and abducted Cal-Mid (Figures 5, 6). It is possible that cluster 1 had a motor behavior that depended more on an adaptation of the distal segment of the foot while keeping the proximal segments in a closer alignment to the neutral position, whereas cluster 2 appears to depend more on the rearfoot and midfoot movements. The alignment of the midfoot and the forefoot in the transverse plane might reflect in the conformation of the transverse arch, since it is defined by the cuboid, cuneiform and metatarsal bones. The smaller deviations of the midfoot in cluster 1 could be related to a lower mobility in this segment and less compliance of the transverse arch. The higher the curvature of the transverse arch the greater the stiffness of the longitudinal arches of the foot, which has an important role during locomotion, especially in providing resistance to the bending in the sagittal plane to favor propulsion (Venkadesan et al., 2020).

These differences in movements in the transverse plane could also be related to the foot progression angle. A toe-out (foot abduction) placement during the stance phase of gait has been shown to increase the forefoot abduction angles (Schallig et al., 2020) and can lead to increased foot pronation during running (Mousavi et al., 2021). However, we did not directly measure the foot progression angle in our study, therefore we cannot confirm this association, and the lack of discriminating features in frontal plane movement between clusters suggests that it is unlikely that inter-cluster differences are solely related to that parameter.

Regarding the effect of the intervention across both clusters, PC1 was the kinematic component that presented a distinct response between clusters, whereby only cluster 1 displayed a difference between baseline and week-8. Runners from cluster 1 reduced the transverse plane deviation of the forefoot, aligning it closer to neutral position, and increased the rearfoot adduction, approximating to the movement pattern observed in cluster 2 after the intervention (Figures 8, 9). Interestingly, this change in cluster 1 was not extended to the Cal-Mid joint, indicating that this subgroup could have a lack of mobility of the midfoot section. The midtarsal joint complex has an important participation in the stance phase and the transverse plane movement is an important component for its function, particularly in the talonavicular joint (Okita et al., 2014; Phan et al., 2019). However, we did not objectively measure the passive mobility of the midfoot in these runners, which hinders a better understanding about the cause of this kinematic behavior, and further investigations are needed.

There were other changes due to the exercise protocol in the sagittal plane for all foot segments, but both clusters responded in the same direction, wherein there was an increase in Sha-Cal plantar flexion, a Cal-Mid dorsiflexion, and a decrease in Mid-Met plantar flexion. This finding shows that both clusters responded to the intervention protocol in a similar way in the sagittal plane, regardless of their distinct kinematic pattern in baseline. This result can be confirmed by a significant decrease in PC3 score in both subgroups {cluster 1 [baseline: 0.9 (11.2); week-8: −4.6 (13.6)]; cluster 2 [baseline: 0.3 (11.6); week-8: −4.9 (10.1)]; ANOVA assessment time effect: *p* = 0.018}, which can be visualized in Figure 9. The adaptability of the foot during weight acceptance appears to be a phenomenon that occurs primarily in the sagittal plane (Blackwood et al., 2005). The fact that both clusters responded to the intervention with changes in the sagittal plane suggests a change in mechanical response to foot loading, which are directly related to the function of the intrinsic foot muscles (Kelly et al., 2014). The foot-core muscles helps counteracting the dorsiflexion moments produced at the midfoot joints (Holowka and Lieberman, 2018) and perhaps the training protocol allowed an increase in their activation, leading to a greater Cal-Mid dorsiflexion and Mid-Met plantar flexion.

Overall, the foot-core exercise intervention promoted biomechanical changes in FA movement patterns in the sagittal and transverse planes, but cluster 1 presented greater responses in the transverse plane than cluster 2. The alignment of the midfoot and the forefoot in the transverse plane might reflect the conformation of the transverse arch. Since cluster 1 presented a midfoot that was more aligned with a neutral position in the transverse plane before the intervention, and responded with an enhance in dorsiflexion after the intervention, one could assume that there was a change in the conformation of the transverse arch that could be related to the exercise protocol.

The clusters did not present differences in demographic or running practice-related variables. Furthermore, there was no difference in injury rates between the identified clusters, indicating that the associations between FA kinematic patterns and running-related injury etiology are not straightforward and still need further investigation. Other studies analyzing lower limb kinematics during running have identified subgroups with different movement patterns in major joints and segments, but did not find a significant association with injuries (Dingenen et al., 2020; Jauhiainen et al., 2020), which corroborates with our results. There was a greater proportion of forefoot strikers in cluster 2, but both subgroups still included runners with both strike patterns, and the kinematic differences between clusters seem to be unrelated to this factor. Midfoot and forefoot strikers have been described to present greater plantar flexion, inversion and adduction of Sha-Cal (Bruening et al., 2018; Deschamps et al., 2019), and more plantar flexion and less inversion of Cal-Mid (Deschamps et al., 2019; Matias et al., 2020). Given that the main between-cluster differences occurred in the transverse plane, other aspects of FA kinematics were probably more determinant for cluster identification and responses to the intervention.

It is important to consider that a PCA was applied to the whole kinematic dataset before performing the HCA, rather than executing it to each joint and plane separately. Although this approach allows the reduced components to represent movement patterns across all joints and planes simultaneously, it increases the dimensionality of the dataset and limits the external validity and stability of the PCA (Jolliffe, 2002). Furthermore, the interpretation of the functional meaning of the components becomes more complex and less detailed within each joint/plane, so the results must be taken with caution. Nevertheless, there is limited variation of the foot joint angles during the stance phase of running, and the analysis of the behavior of the whole kinematic chain allows the identification of combinations between joints and planes in the movement pattern, which can be important in a segment that is composed by multiple joints. Still, we reiterate that we may not be able to extrapolate our results to the population, since we had a relatively small sample size for the application of a PCA and an HCA.

A few aspects of the methods for kinematic data acquisition should also be considered in this study, since the participants ran on a treadmill, conditions that were different from their usual practice. Nevertheless, since kinematic patterns of the lower limbs have already been shown to be similar between overground and treadmill running (Fellin et al., 2010), we believe that the results in this study represent the patterns adopted by the subjects during their regular running routine. It is also important to emphasize that analysis on the effects of the intervention had an exploratory purpose and a proper conclusion about the response to a foot-core exercise program can only be reached with the inclusion of the week-8 assessment of the control group in the analysis, which unfortunately were not available for this study.

The majority of randomized controlled trials aim to describe the general effect of a treatment on some outcome, and the findings are important to inform clinical practice and policy decisions. However, usually the treatment effect is heterogeneous across the population because of individual variations. In this context, understanding which subgroups of individuals may be more or less likely to benefit from a treatment protocol is important for providing evidence for clinical decisions (Lesko et al., 2018). For example, lower limb kinematics during locomotor tasks have been shown to be related to the responsiveness to exercise-based treatment in individuals with knee osteoarthritis (Kobsar et al., 2015) and patellofemoral pain (Watari et al., 2016). Seeing that the intervention protocol in this study was effective in reducing the incidence of running-related injuries (Taddei et al., 2020a), the results of this study can help understanding how individual variations of baseline motor behaviors can influence the response to these exercises and this approach can help guiding treatment strategies that are more individual-centered, taking into account their initial mechanical patterns. Future studies should investigate whether the identified movement patterns could be influencing the protective effects against running-related injuries that this exercise program has presented, by stratifying the experimental groups based on kinematic patterns.

We identified two clusters of FA movement patterns during running among asymptomatic recreational runners that differed mainly in transverse plane movements of foot segments, with one cluster presenting a more pronounced forefoot abduction, while the other displayed greater deviations from neutral position of the rearfoot (greater adduction) and midfoot (greater abduction). After a foot-core exercise-based intervention, runners that presented transverse plane deviations in the distal segments at baseline, responded distinctly to the program, increasing the rearfoot adduction and moving the forefoot closer to neutral position during running. This study suggests that the individual foot biomechanical pattern can influence the response to exercise interventions and this approach can help guiding the development of individual-centered treatment strategies.

## Data Availability Statement

The raw data supporting the conclusions of this manuscript can be accessed through the following data repository link: https://doi.org/10.6084/m9.figshare.13484715.v2.

## Ethics Statement

The studies involving human participants were reviewed and approved by Ethics Committee of the School of Medicine of the University of São Paulo approval # CAAE 41171215.7.0000.0065. The patients/participants provided their written informed consent to participate in this study.

## Author Contributions

RW was the main author of this manuscript, being responsible for its conceptualization, data processing and analysis, and writing of the original draft. ES participated in the conceptualization of the study and in the interpretation of the results. JS participated in the data processing and curation and in the interpretation of results. AM and UT were responsible for the execution of the clinical trial, and data acquisition and processing. IS supervised and administered the research project, being responsible for funding acquisition, study conceptualization, and made contributions to the manuscript review and editing. All authors have made substantial contributions to the manuscript, and they all read, provided feedback and approved the submitted version.

## Conflict of Interest

The authors declare that the research was conducted in the absence of any commercial or financial relationships that could be construed as a potential conflict of interest.

## Abbreviations

Cal-Mid: angle between calcaneus and midfoot
CCC: cophenetic correlation coefficient
FA: foot-ankle complex
HCA: hierarchical cluster analysis
Mid-Met: angle between midfoot and metatarsal bones
PC: principal component
PCA: principal component analysis
Sha-Cal: angle between shank and calcaneus.

## References

[B1] Abdi H. Williams L. J. (2010). Principal component analysis. *Wiley Interdiscip. Rev. Comput. Stat.* 2 433–459. 10.1002/wics.101

[B2] Arbelaitz O. Gurrutxaga I. Muguerza J. Pérez J. M. Perona I. (2013). An extensive comparative study of cluster validity indices. *Pattern Recognition* 46 243–256. 10.1016/j.patcog.2012.07.021

[B3] Blackwood C. B. Yuen T. J. Sangeorzan B. J. Ledoux W. R. (2005). The midtarsal joint locking mechanism. *Foot Ankle Int.* 26 1074–1080. 10.1177/107110070502601213 16390642

[B4] Brandon S. C. E. Graham R. B. Almosnino S. Sadler E. M. Stevenson J. M. Deluzio K. J. (2013). Interpreting principal components in biomechanics: Representative extremes and single component reconstruction. *J. Electromyogr. Kinesiol.* 23 1304–1310. 10.1016/j.jelekin.2013.09.010 24209874

[B5] Bruening D. A. Pohl M. B. Takahashi K. Z. Barrios J. A. (2018). Midtarsal locking, the windlass mechanism, and running strike pattern: A kinematic and kinetic assessment. *J. Biomech.* 73 185–191. 10.1016/j.jbiomech.2018.04.010 29680311 PMC5944854

[B6] Carvalho P. R. Munita C. S. Lapolli A. L. (2019). Validity studies among hierarchical methods of cluster analysis using cophenetic correlation coefficient. *Braz. J. Radiat. Sci.* 7 1–14. 10.15392/bjrs.v7i2a.668

[B7] Deschamps K. Eerdekens M. Peters H. Matricali G. A. Staes F. (2019). Multi-segment foot kinematics during running and its association with striking patterns. *Sport. Biomech.*, 1–14. 10.1080/14763141.2019.1645203 31464161

[B8] Dingenen B. Staes F. Vanelderen R. Ceyssens L. Malliaras P. Barton C. J. (2020). Subclassification of recreational runners with a running-related injury based on running kinematics evaluated with marker-based two-dimensional video analysis. *Phys. Ther. Sport* 44 99–106. 10.1016/j.ptsp.2020.04.032 32504962

[B9] Fellin R. E. Manal K. Davis I. S. (2010). Comparison of lower extremity kinematic curves during overground and treadmill running. *J. Appl. Biomech.* 26 407–414. 10.1123/jab.26.4.407 21245500 PMC3266869

[B10] Goldmann J.-P. Sanno M. Willwacher S. Heinrich K. Brüggemann G.-P. (2013). The potential of toe flexor muscles to enhance performance. *J. Sports Sci.* 31 424–433. 10.1080/02640414.2012.736627 23106289

[B11] Holowka N. B. Lieberman D. E. (2018). Rethinking the evolution of the human foot: Insights from experimental research. *J. Exp. Biol.* 221 jeb174425. 10.1242/jeb.174425 30190415

[B12] Jauhiainen S. Pohl A. J. Äyrämö S. Kauppi J. P. Ferber R. (2020). A hierarchical cluster analysis to determine whether injured runners exhibit similar kinematic gait patterns. *Scand J Med Sci Sports* 30 732–740. 10.1111/sms.13624 31900980

[B13] Jolliffe I. T. (2002). *Principal component analysis*, 2nd Edn. New York, NY: Springer.

[B14] Kelly L. A. Cresswell A. G. Farris D. J. (2018). The energetic behaviour of the human foot across a range of running speeds. *Sci. Rep.* 8 1–6. 10.1038/s41598-018-28946-1 30002498 PMC6043578

[B15] Kelly L. A. Cresswell A. G. Racinais S. Whiteley R. Lichtwark G. (2014). Intrinsic foot muscles have the capacity to control deformation of the longitudinal arch. *J. R. Soc. Interface* 11 20131188. 10.1098/rsif.2013.1188 24478287 PMC3928948

[B16] Ker R. F. Bennett M. B. Bibby S. R. Kester R. C. Alexander R. M. (1987). The spring in the arch of the human foot. *Nature* 325 147–149. 10.1038/325147a0 3808070

[B17] Kettaneh N. Berglund A. Wold S. (2005). PCA and PLS with very large data sets. *Comput. Stat. Data Anal.* 48 69–85. 10.1016/j.csda.2003.11.027

[B18] Kobsar D. Osis S. T. Hettinga B. A. Ferber R. (2015). Gait Biomechanics and Patient-Reported Function as Predictors of Response to a Hip Strengthening Exercise Intervention in Patients with Knee Osteoarthritis. *PLoS One* 10:e0139923. 10.1371/journal.pone.0139923 26444426 PMC4596804

[B19] Leardini A. Benedetti M. G. Berti L. Bettinelli D. Nativo R. Giannini S. (2007). Rear-foot, mid-foot and fore-foot motion during the stance phase of gait. *Gait Posture* 25 453–462. 10.1016/J.GAITPOST.2006.05.017 16965916

[B20] Lesko C. R. Henderson N. C. Varadhan R. (2018). Considerations when assessing heterogeneity of treatment effect in patient-centered outcomes research. *J. Clin. Epidemiol.* 100 22–31. 10.1016/j.jclinepi.2018.04.005 29654822 PMC6467652

[B21] Macera C. A. Pate R. R. Powell K. E. Jackson K. L. Kendrick J. S. Craven T. E. (1989). Predicting Lower-Extremity Injuries Among Habitual Runners. *Arch. Intern. Med.* 149 2565. 10.1001/archinte.1989.00390110117026 2818115

[B22] Matias A. B. Caravaggi P. Taddei U. T. Leardini A. Sacco I. C. N. (2020). Rearfoot, Midfoot, and Forefoot Motion in Naturally Forefoot and Rearfoot Strike Runners during Treadmill Running. *Appl. Sci.* 10 7811. 10.3390/app10217811

[B23] Matias A. B. B. Taddei U. T. T. Duarte M. Sacco I. C. N. C. N. (2016). Protocol for evaluating the effects of a therapeutic foot exercise program on injury incidence, foot functionality and biomechanics in long-distance runners: a randomized controlled trial. *BMC Musculoskelet. Disord.* 17:160. 10.1186/s12891-016-1016-9 27075480 PMC4831173

[B24] McKeon P. O. Hertel J. Bramble D. Davis I. (2015). The foot core system: a new paradigm for understanding intrinsic foot muscle function. *Br. J. Sports Med.* 49 290. 10.1136/bjsports-2013-092690 24659509

[B25] Mickle K. J. Caputi P. Potter J. M. Steele J. R. (2016). Efficacy of a progressive resistance exercise program to increase toe flexor strength in older people. *Clin. Biomech.* 40 14–19. 10.1016/j.clinbiomech.2016.10.005 27780109

[B26] Mickle K. J. Munro B. J. Lord S. R. Menz H. B. Steele J. R. (2009). ISB Clinical Biomechanics Award 2009: Toe weakness and deformity increase the risk of falls in older people. *Clin. Biomech.* 24 787–791. 10.1016/J.CLINBIOMECH.2009.08.011 19751956

[B27] Mousavi S. H. Van Kouwenhove L. Rajabi R. Zwerver J. Hijmans J. M. (2021). The effect of changing foot progression angle using real-time visual feedback on rearfoot eversion during running. *PLoS One* 16:e0246425. 10.1371/journal.pone.0246425 33566828 PMC7875396

[B28] Nisha P. J. K. Kaur P. J. (2016). “Cluster quality based performance evaluation of hierarchical clustering method,” in *Proceedings of the 1st International Conference on Next Generation Computing Technologies, NGCT 2015*, (New York, NY: Institute of Electrical and Electronics Engineers Inc), 649–653. 10.1109/NGCT.2015.7375201

[B29] Okita N. Meyers S. A. Challis J. H. Sharkey N. A. (2014). Midtarsal joint locking: New perspectives on an old paradigm. *J. Orthop. Res.* 32 110–115. 10.1002/jor.22477 24038197

[B30] Phan C. B. Shin G. Lee K. M. Koo S. (2019). Skeletal kinematics of the midtarsal joint during walking: Midtarsal joint locking revisited. *J. Biomech.* 95 109287. 10.1016/j.jbiomech.2019.07.031 31431345

[B31] Phinyomark A. Osis S. Hettinga B. A. Ferber R. (2015). Kinematic gait patterns in healthy runners: A hierarchical cluster analysis. *J. Biomech.* 48 3897–3904. 10.1016/j.jbiomech.2015.09.025 26456422

[B32] Portinaro N. Leardini A. Panou A. Monzani V. Caravaggi P. (2014). Modifying the Rizzoli foot model to improve the diagnosis of pes-planus: Application to kinematics of feet in teenagers. *J. Foot Ankle Res.* 7 1–7. 10.1186/s13047-014-0057-2 25558289 PMC4282742

[B33] Riddick R. Farris D. J. Kelly L. A. (2019). The foot is more than a spring: human foot muscles perform work to adapt to the energetic requirements of locomotion. *J. R. Soc. Interface* 16 20180680. 10.1098/rsif.2018.0680 30958152 PMC6364639

[B34] Rohlf F. J. (1970). Adaptive Hierarchical Clustering Schemes. *Syst. Biol.* 19 58–82. 10.1093/sysbio/19.1.58

[B35] Rousseeuw P. J. (1987). Silhouettes: A graphical aid to the interpretation and validation of cluster analysis. *J. Comput. Appl. Math.* 20 53–65. 10.1016/0377-0427(87)90125-7

[B36] Schallig W. van den Noort J. C. McCahill J. Stebbins J. Leardini A. Maas M. (2020). Comparing the kinematic output of the Oxford and Rizzoli Foot Models during normal gait and voluntary pathological gait in healthy adults. *Gait Posture* 82 126–132. 10.1016/j.gaitpost.2020.08.126 32920448

[B37] Sokal R. R. Rohlf F. J. (1962). The comparison of dendrograms by objective methods. *Taxon* 11 33–40. 10.2307/1217208

[B38] Taddei U. T. Matias A. B. Duarte M. Sacco I. C. N. (2020a). Foot Core Training to Prevent Running-Related Injuries: A Survival Analysis of a Single-Blind, Randomized Controlled Trial. *Am. J. Sports Med* 48 3610–3619. 10.1177/0363546520969205 33156692

[B39] Taddei U. T. Matias A. B. Ribeiro F. I. A. Bus S. A. Sacco I. C. N. (2020b). Effects of a foot strengthening program on foot muscle morphology and running mechanics: A proof-of-concept, single-blind randomized controlled trial. *Phys. Ther. Sport* 42 107–115. 10.1016/j.ptsp.2020.01.007 31962191

[B40] Trujillo-Ortiz A. (2020). *moutlier1.* Available online at: www.mathworks.com/matlabcentral/fileexchange/12252-moutlier1 (accessed May 19, 2020).

[B41] Venkadesan M. Yawar A. Eng C. M. Dias M. A. Singh D. K. Tommasini S. M. (2020). Stiffness of the human foot and evolution of the transverse arch. *Nature* 579 97–100. 10.1038/s41586-020-2053-y 32103182

[B42] Ward J. H. (1963). Hierarchical Grouping to Optimize an Objective Function. *J. Am. Stat. Assoc.* 58 236–244. 10.1080/01621459.1963.10500845

[B43] Watari R. Kobsar D. Phinyomark A. Osis S. Ferber R. (2016). Determination of patellofemoral pain sub-groups and development of a method for predicting treatment outcome using running gait kinematics. *Clin. Biomech.* 38 13–21. 10.1016/j.clinbiomech.2016.08.003 27522485

[B44] Wu G. Siegler S. Allard P. Kirtley C. Leardini A. Rosenbaum D. (2002). ISB recommendation on definitions of joint coordinate system of various joints for the reporting of human joint motion–part I: ankle, hip, and spine. *International Society of Biomechanics. J. Biomech.* 35 543–548. 10.1016/S0021-9290(01)00222-6 11934426

[B45] Zifchock R. A. Davis I. Hillstrom H. Song J. (2006). The effect of gender, age, and lateral dominance on arch height and arch stiffness. *Foot Ankle Int.* 27 367–372. 10.1177/107110070602700509 16701058

